# Differences in heart rate variability and body composition in breast cancer survivors and women without cancer

**DOI:** 10.1038/s41598-021-93713-8

**Published:** 2021-07-14

**Authors:** Daniel Escutia-Reyes, José de Jesús Garduño-García, Gerardo Emilio-López-Chávez, Ángel Gómez-Villanueva, Adriana Cristina Pliego-Carrillo, Alexandra Estela Soto-Piña, José Javier Reyes-Lagos

**Affiliations:** 1 School of Medicine, Autonomous University of Mexico State (UAEMéx), State of Mexico 50180 Toluca, Mexico; 2 Regional General Hospital No. 251, Mexican Institute of Social Security (IMSS), State of Mexico 52148 Metepec, Mexico

**Keywords:** Biomedical engineering, Breast cancer, Diagnostic markers, Cancer, Computational biology and bioinformatics, Neuroscience, Physiology, Biomarkers, Health care

## Abstract

The aim of this study was to explore cardiac autonomic changes assessed by linear and nonlinear indexes of heart rate variability (HRV) and body composition modifications in breast cancer survivors and cancer-free control women. Women who were breast cancer survivors (BCS, *n* = 27) and without cancer with similar characteristics (Control, *n* = 31) were recruited for this study. We calculated some relevant linear and nonlinear parameters of 5 min of RR interval time series such as mean RR interval (RR_ave_), the corrected Poincaré index (cSD1/SD2), the sample entropy (SampEn), the long-term fractal scaling exponent (α_2_) and 2UV from symbolic dynamics. Additionally, we indirectly assessed body composition measures such as body weight, fat mass, visceral fat rating (VFR), normalized VRF (nVFR), muscle mass, metabolic age, and total body water. We found that diverse HRV indexes and only one body composition measure showed statistical differences (*p* < 0.05) between the BCS and Control groups. RR_ave_: 729 (648–802) vs. 795 (713–852) ms; cSD2/SD1: 3.4 (2.7–5.0) vs. 2.9 (2.3–3.5); SampEn: 1.5 (1.3–1.8) vs. 1.7 (1.5–1.8); α_2_: 0.6 (0.3–0.6) vs. 0.5 (0.4–0.5); 2UV: 7.1 (4.3–11.5) vs. 10.8 (6.4–15.7) and nVFR 0.12 (0.11–0.13) vs. 0.10 (0.08–0.12) points/kg, respectively. The nVFR was strongly significantly correlated with several indexes of HRV only in the BCS group.Our findings suggest that BCS exhibit lower parasympathetic cardiac activity and changes in HRV patterns compared to Controls. A concomitant increase of visceral fat, among other factors, may contribute to cardiac autonomic disturbances and changes in HRV patterns in BCS.

## Introduction

Breast cancer is among the five most common forms of cancer, and it is one of the most important causes of death worldwide, accounting for an estimated 627,000 deaths in 2018^1^. In Latin America, breast cancer ranks as the first cancer type among women regarding new cases and deaths^2^. These statistics become more relevant in public health since breast cancer can lead to associated diseases and alterations both in early stages and even years after treatment and recuperation. Potential factors, such as metabolic dysregulation and weight gain, are associated with autonomic dysfunction and increased cardiovascular disease risk in patients with breast cancer^3^. Different studies indicate that breast cancer survivors (BCS) show a stronger association with metabolic syndrome^4^, diabetes^5^, and abdominal obesity^6^, which are major risk factors for cardiovascular disease^7^.

Taking into account that metabolic modifications are present in BCS, body composition seems to be a factor of interest in breast cancer management, specifically at the tissue level witch refers to amounts and distributions of adipose, skeletal, and muscle tissue in the patients^8,9^. Interestingly, the study of body composition has been considered as one of the most promising areas in oncology^10^. Concerning breast cancer, evidence suggests that body composition is a crucial contributor to clinical outcomes after surgery^11^. In addition, relevant findings indicate that changes in body composition may lead to modifications in the autonomic cardiac function^12^. It is known that autonomic dysfunction represents a loss of normal autonomic control of the cardiovascular system associated with both increases in sympathetic nervous system activation and reduced efficacy of the parasympathetic nervous system^3^ that may affect one or more systems of the body^13,14^.

Recent studies have suggested that an altered cardiac autonomic activity is associated with a high share of adipose tissue^15^. Other findings have also indicated that BCS may also show an autonomic impairment due to adjuvant therapies^16^, among other factors. Notably, BCS exhibit numerous dysfunctions: lower parasympathetic cardiac function, lower aerobic fitness, signs of altered metabolism, and higher perception of fatigue^17^. Data indicate that physical training can reverse impaired cardiorespiratory fitness and autonomic modulation in women with breast cancer receiving adjuvant therapy^18^.

The heart rate variability (HRV) analysis has been established as a complementary non-invasive and economical tool for the early diagnosis and better prognosis of autonomic cardiac dysfunction and survival in BCS women^19^. Studies have shown that the presence of a cardiovascular imbalance in BCS compared to healthy controls suggests that traditional linear indexes of HRV study could be clinically valuable for detecting cardiovascular disease in BCS^20^. Nevertheless, linear HRV analysis techniques are often insufficient to characterize the complex dynamics of the heartbeat generation since the mechanisms involved in cardiovascular regulation probably interact with each other in a nonlinear way^21^. With this consideration, promising nonlinear tools such as symbolic dynamics and fractal indexes have been introduced to describe the complexity of HRV. Both methodologies are especially useful given that minimize the effects of non-stationarities^22,23^. The nonlinear methods have shown great promise in the detection and diagnosis of heart failure^24^ and the evaluation of physical condition and well-being during physical activity tests^25^. However, in BCS, the nonlinear properties of cardiac dynamics related to changes in the autonomic nervous system (ANS) and complexity have not been fully elucidated.

As far as we know, no other studies have compared autonomic cardiac activity assessed by linear and nonlinear HRV measures in conjunction with various measures of body composition values between BCS and cancer-free women. With this background, this study aims to assess the autonomic cardiac function by nonlinear features of heart fluctuations, and body composition modifications in BCS and women with no cancer diagnosis under similar characteristics. We hypothesized that women who are BCS manifest cardiac autonomic disturbances characterized by a diminished vagal tone related to changes in body composition.

## Methods

### Study design

In this preliminary cross-sectional study, Mexican women between 30 and 67 years old who attended the Oncology Service at the Regional General Hospital No. 251 of the Mexican Institute of Social Security (Metepec, State of Mexico, Mexico), from March 2019 until July 2019 were invited to participate in this study. The BCS group included women who were in their post-cancer follow-up appointment. The inclusion criteria included the following: (a) previous diagnosis of breast cancer; (b) women who had undergone breast cancer surgery (lumpectomy or mastectomy) anywhere from 1 to 5 years earlier and women who did not require surgery; (c) no evidence of metastases, (d) absence of respiratory and cardiovascular diseases, diabetes mellitus, thyroid dysfunction, and hypertension; (e) normotensive; (f) capacity to stand up unaided, and (g) capacity to answer a clinical interview, including a familiar history of cancer. The exclusion criteria consisted of the following: (a) women under anticancer medication (e.g., tamoxifen^26^) or any other medication at the moment of the study; (b) women undergoing chemotherapy or radiotherapy (c) women being treated with any other adjuvant therapy, (d) super-obese women (BMI > 50 kg/m^2^) and (d) smoking women. The elimination criteria involved women whose QRS complex was not feasible to detect due to excessive movement artifacts or low signal voltage.

Moreover, the control group included women without cancer diagnosis with similar characteristics to the BCS group (ethnicity, age, weight, BMI, and height). In all controls, the presence of chronic diseases or pharmacological treatment was excluded by history and standard medical examination.

The sample size estimation was based on the study of Romanholi Palma et al.^27^ and was determined using the G*Power software^28^. We considered an 80% test power, an alpha error of 5% for a one-tailed test. The minimum sample size was determined to be 14 participants per group. Specifically, a one-tailed t-test was selected for this preliminary study based on the directional hypothesis that vagal tone is diminished in BCS.

### Electrocardiogram recording and preprocessing

On the day of the study, all participants arrived in the Oncology service, having avoided caffeinated or alcoholic beverages. Electrophysiological recordings were performed between 08:00 a.m. and 12:00 p.m. to account for circadian rhythms of the heartbeat. All participants were asked to relax and record in a standard seated position at rest^29^. The first lead (DI) of the electrocardiogram (ECG) was recorded for 5 min by using an ECG sensor model EKG-BTA (Vernier^®^, Beaverton, Oregon, USA) for NI Elvis II (Texas Instruments^®^, Dallas, Texas, USA) and superficial disposable electrodes. Electrocardiographic data were acquired with a PC at a sampling rate of 1000 Hz using the Biosignal Logger and Player software (National instruments^®^, Austin, Texas, USA).

Raw ECG recordings for both BCS and Control groups were then processed using previously validated algorithms to generate RR time series^30^. All the RR time series were reconditioned by a filtering approach to exclude possible artifacts such as ectopic beats, arrhythmic events, and noise effects that may alter the estimation of the HRV^31^. The reconditioning step includes 60 Hz filtering for line noise elimination and an adaptative filter for artifact correction. All these calculations were obtained using Matlab^®^ software (the MathWorks, Inc. Natick, Massachusetts, USA).

### Heart rate variability (HRV) assessment

We assessed HRV according to methodological standards proposed by the Task Force of the European Society of Cardiology and the North American Society of Pacing and Electrophysiology for HRV^32^. We used the Kubios software version 3.1 (Kuopio, Finland)^25^ to analyze some linear and nonlinear measures of the RR time series. The following linear (time-domain) indexes were included: the mean RR interval (RR_ave_), the standard deviation of the RR time series of normal sinus beats (SDNN, a biomarker of global HRV), the root mean square of successive differences (RMSSD), and the percentage of pairs of successive RR intervals that differ by more than 50 ms, these last two biomarkers are associated to the cardiac parasympathetic function^32^.

Linear (frequency-domain) indexes were also reported, such as the normalized high-frequency (HFnu: 0.15–0.4 Hz). HFnu is a parasympathetic dominance index^33^. Also, we included a marker of respiratory sinus arrhythmia, quantified as the power of HRV in the high-frequency band (HF: 0.15–0.4 Hz) expressed in absolute units (HF ms^2^), being an index for vagal modulation of heart rate^34,35^.

By default, a 4 Hz interpolation was set in the Kubios software. The spectrum for the selected RR time series was computed with Welch's periodogram method (FFT spectrum). Since our signal length is five minutes, we adjusted the window to 128 s with a 50% overlap to have enough windows to estimate the spectral components correctly^36^.

Furthermore, we performed a nonlinear analysis of HRV. The selection of nonlinear indexes was based on the findings carried out by Maestri et al., who has reported indexes that are not redundant among themselves and provide valuable predictive information^37^. This study assessed four of the six major families of some nonlinear indexes: Poincaré plots, fractality, entropy, and symbolic dynamics.

Concerning Poincaré plots, we considered the indexes SD1, SD2, and SD1/SD2. SD1 is an index of short-term variability and reflects parasympathetic activity, while the SD2 index measures long-term variability and reflects the overall variability. The (SD1/SD2) ratio represents the balance between long- and short-term HRV^38^. The quantitative analysis of a plot involves fitting an ellipse to the Poincaré plot, which corresponds to the length of the minor (SD1) and major (SD2) axes.

We considered the following fractal indexes from the detrended fluctuation analysis (DFA) method: the short-term fractal exponent (α_1_), corresponding to 4–11 beats, and the long-term fractal exponent (α_2_), ranging from 11 to N/4 beats^39^. When α = 0.5, there is no correlation, and the time series shows white noise behavior; if α = 1.5, the time series resemble Brownian motion, and if it is 0.5 < α < 1.5, there are positive correlations. If α ≈ 1.0 indicates a fractal-like behavior, if it reaches values above 1.0, the system tends to be less complex and linear^40^.

The fact that HRV is dependent on mean RR interval (or mean heart rate) is widely recognized. A higher mean RR interval naturally leads to higher variability. In considering this fact, some indexes were normalized (or corrected) to attenuate the mathematical HRV dependence on heart rate^41^ based on the method proposed by van Roon^42^ and recommended by de Geus^43^. Thus, the following indexes were corrected: RMSSD, SDNN, SD1, SD2, and SD1/SD2 ratio, generating the normalized indexes cRMSSD, cSDNN, cSD1, cSD2, and cSD1/SD2 ratio, respectively. The mentioned method for normalization is exemplified in Eq. (1) for the RMSSD. It applies similarly to the rest of the corrected indexes.

1 $$cRMSSD = 1000 \times \frac{{RMSSD}}{{RRave}}.$$

Similarly, the spectral index HF (ms^2^) was corrected according to Sacha et al.^41^ by dividing the original HF value by RR_ave_ squared, thus obtaining cHF.

Regarding the entropy family used to assess the regularity/irregularity of the RR time series, we estimated the sample entropy (SampEn) calculated with *m *= 2 and *r* = 0.2, as described by Richman and Moorman^44^.

Finally, the symbolic dynamics family was evaluated using the PyBioS freeware^45^ considering 0V, 1V, 2LV, 2UV indexes computed from the sigma method. This family of indexes evaluates the activity of both autonomous branches even when their modulation is not reciprocal^46^. Symbolic analysis reveals the parasympathetic and sympathetic and modulations on the heart exclusively by the indexes 2UV and 0V, respectively. A full description of this technique is described in Ref.^47^.

### Body composition estimation

Bioelectrical impedance analysis (BIA) is a non-invasive, low-cost, helpful, and validated tool for estimating body composition^48^. The analysis is achieved by measuring the bioimpedance of an electrical current transmitted to the body through electrodes placed on the feet^49^. On the day of the study, a body composition analyzer, which employed BIA, was used to estimate body composition (BC-533 InnerScan Body Composition Monitor^®^, Tanita Corp., Itabashi-Ku Tokyo, Japan). Firstly, the heights of all subjects were measured and recorded. Then, participants were weighed, and body composition values were indirectly estimated using the device. The following body composition measures were collected for the BCS and Control groups: body fat percentage, body water percentage, muscle mass, bone mass, predicted daily calorie intake (DCI), metabolic age, and visceral fat rating (VFR). Particularly, VFR is given as a specific rating: (0–59 points). Ratings from 1 to 12 points indicate that the subject has a healthy level of visceral fat, while ratings from 13 to 59 points indicate that the subject has an excess level of visceral fat. The visceral fat rating has been widely applied in medical research as an indirect visceral fat amount in females^50^ and mixed-gender groups^51^. We calculated the normalized visceral fat rating (nVFR) by dividing the visceral fat rating by each participant's body weight.

### Ethical considerations

This study was approved by the Research Ethics Committee No. 1503 from the Regional General Hospital No. 251 of the Mexican Institute of Social Security (IMSS). The Federal Commission for Protection Against Health Risks (COFEPRIS) authorizes this committee (authorization no. 17 CI 15 104 037) and the National Research National Commission of Bioethics in Mexico (CONABIO, authorization no. 15 CEI 002 2017033). This protocol was registered under the code R-2019-1503-012. All volunteers in this study signed an Informed Consent Form when they agreed to participate, and all methods were performed in accordance with the relevant guidelines and regulations.

### Statistical analysis

The statistical analysis was done with GraphPad Prism version 8.00 for Windows (GraphPad Software, La Jolla, California USA). Descriptive results were presented as median (25th–75th percentile) for quantitative variables and frequency (percentage) for categorical variables. A Shapiro–Shapiro–Wilk test was used to assess the normality of distribution. However, the data did not appear to have a normal distribution. Thus, the continuous variables were compared using one-tailed Mann–Whitney's U tests, and categorical variables were evaluated by Fisher's exact test. Associations between body composition and HRV measures (BCS group) were evaluated by Spearman's correlation coefficient. For all tests, results of *p* < 0.05 were considered significant.

## Results

Of 80 invited BCS women, 20 women were unwilling to participate in the study; 32 women were excluded because they were under chemotherapy, radiotherapy, or medication. Finally, the data of 27 BCS women were analyzed. Thirty-one women without cancer conformed the Control group with similar characteristics to the BCS group. Table 1 shows their general clinical parameters; as was expected, there were no significant differences between both groups (*p* > 0.05).

**Table 1:** General clinical characteristics of the study population.

	BCS n = 27	Control N = 31	Significance *p*
Age (years)	56 (46–62)	51 (43–56)	0.11
Height (cm)	152 (148–157)	155 (150–158)	0.38
Weight (kg)	65.0 (55.4–76.3)	67.3 (58.2–69.8)	0.83
BMI (kg/m^2^)	27.5 (24.7–32.4)	27.4 (24.9–37.4)	0.39
Menarche (years)	12 (12–13)	13 (12–14)	0.48
Number of gestations	2 (1–3)	3 (2–4)	0.13
Time since initial diagnose of cancer (years)	3.8 (2.4–6.0)	–	–
Time since surgery (years)	3.7 (1.4–4.8)	–	–
Lumpectomy/mastectomy	24 (88.8)	–	–
Family history of cancer (yes)	11 (40.7)	8 (25.8)	0.17
Family history of cancer (no)	16 (59.3)	23 (74.2)	
Smoking	0 (0)	0 (0)	

Data as presented as median (25th, 75th percentiles) or numbers (%). Mann–Whitney U test (continuous variables) between Breast cancer survivors (BCS) and Control groups. Fisher's exact test for categorical variables.

Table 2 exhibits the HRV linear and nonlinear indexes, several significant differences (*p* < 0.05) were found between the BCS and Control groups. RR_ave_: 729 (648–802) vs. 795 (713–852) ms; cSD2/SD1: 3.4 (2.7–5.0) vs. 2.9 (2.3–3.5); SampEn: 1.5 (1.3–1.8) vs. 1.7 (1.5–1.8); α_2_ 0.6 (0.3–0.6) vs 0.5 (0.4–0.5); 2UV: 7.1 (4.3–11.5) vs. 10.8 (6.4–15.7). Notably, the cSD2/SD1 ratio followed by the RR_ave_, and 2UV had lower p-values than other HRV indexes.

**Table 2:** Comparison of linear and nonlinear heart rate variability (HRV) indexes between the Breast Cancer Survivors (BCS) and Control groups.

	BCS n = 27	Control n = 31	Significance *p*
**RR**_**ave**_** (ms)**	**729 (648–802)**	**795 (713–852)**	**0.01**
cSDNN	32.2 (20.8–43.7)	32.1 (24.6–44.2)	0.25
cRMSSD	19.6 (11.8–33.4)	23.7 (18.3–36.1)	0.07
pNN50 (%)	0.5 (0.0–5.6)	1.5 (0.0–7.7)	0.12
HFnu	19.9 (12.9–28.9)	19.9 (18.2–29.8)	0.07
cHF	0.14 (0.04–0.43)	0.16 (0.11–0.35)	0.18
cSD1	13.8 (8.3–23.6)	16.7 (13.0–25.5)	0.07
cSD2	42.6 (28.3–59.3)	41.5 (31.7–55.2)	0.31
**cSD2/SD1**	**3.4 (2.7–5.0)**	**2.9 (2.3–3.5)**	**0.001**
**SampEn**	**1.5 (1.3–1.8)**	**1.7 (1.5–1.8)**	**0.04**
α_1_	1.3 (1.2–1.5)	1.2 (1.1–1.4)	0.05
**α**_**2**_	**0.6 (0.3–0.6)**	**0.5 (0.4–0.5)**	**0.04**
0V (%)	52.2 (39.9–66.8)	46.42 (38.4–53.6)	0.05
1V (%)	38.1 (28.61–41.5)	39.4 (36.7–43.7)	0.05
2LV (%)	1.8 (0.0–6.9)	1.7 (0.9–3.3)	0.37
**2UV (%)**	**7.1 (4.3–11.5)**	**10.8 (6.4–15.7)**	**0.03**

Data are presented as median (25th, 75th percentiles). Mann–Whitney's U tests between BCS and Control groups. Bold values correspond to significant results (*p *value < 0.05).

Table 3 depicts all the body composition measures indirectly estimated by BIA. Interestingly, only one body composition measure showed statistical differences (*p* < 0.05) between the BCS and Control groups: nVFR: 0.12 (0.11–0.13) vs. 0.10 (0.08–0.12) points/kg, respectively.

**Table 3:** Comparison of body composition measures between Breast Cancer Survivors (BCS) and control groups.

	BCS n = 27	Control N = 31	Significance *p*
Body fat (%)	35.5 (29.8–40.4)	34.8 (29.0–39.1)	0.24
Water (%)	44.5 (41.9–47.3)	45.2 (42.5–48.4)	0.16
VFR (points)	9 (6–10)	7 (6–9)	0.07
**nVFR (points/kg)**	**0.12 (0.11–0.13)**	**0.10 (0.08–0.12)**	**< 0.01**
Muscle mass (kg)	39.6 (33.6–42.7)	40.3 (38.3–42.0)	0.18
Bone mass (kg)	2.1 (2.0–2.3)	2.2 (2.1–2.3)	0.13
DCI (kcal)	1990 (1819–2146)	2008 (1891–2119)	0.31
Metabolic age (years)	49 (34–50)	49 (32–50)	0.43

*VFR* visceral fat rating, *nVFR* normalized visceral fat rating, *DCI* daily caloric intake.

Data as presented as median (25th, 75th percentiles). Mann–Whitney's U tests between BCS and control groups. Bold values correspond to significant results (*p* value < 0.05).

We explored associations between body composition measures and HRV indexes for both groups. Although the mentioned correlations were executed for both groups, only BCS presented significant correlations, showing strong positive and negative associations for nVFR. Table 4a depicts the significant (*p* < 0.05) Spearman's rank correlation coefficient (ρ) between nVFR and HRV variables for BCS. Notably, BCS's abdominal fat is linked with multiple autonomic and nonlinear features variables. Table 4b exhibits the analogous correlations between nVFR and HRV for the Control group for comparison purposes.

**Table 4:** Spearman correlations between normalized visceral fat rating (nVFR) and linear and nonlinear HRV indexes in (a) breast cancer survivors (BCS) and (b) Control group.

BCS	pNN50	α_1_	α_2_	0V	1V	2LV	2UV	cHF	HFnu	cRMSSD	cSD1	cSD2	cSD2/SD1	cSDNN
**(a)**														
ρ	− 0.5893	0.4952	0.5342	0.5459	− 0.5245	− 0.6085	− 0.5941	− 0.5372	− 0.4506	− 0.5934	− 0.5934	− 0.5745	0.4389	− 0.6203
*p* value	0.0006	0.0043	0.0021	0.0016	0.0025	0.0004	0.0005	0.0019	0.0092	0.0006	0.0006	0.0009	0.0110	0.0003

Control	pNN50	α_1_	α_2_	0V	1V	2LV	2UV	cHF	HFnu	cRMSSD	cSD1	cSD2	cSD2/SD1	cSDNN
**(b)**														
ρ	− 0.1188	− 0.0326	0.1309	0.0395	0.0145	− 0.2784	0.1057	− 0.2501	− 0.0728	− 0.1565	− 0.1565	− 0.2535	− 0.0796	− 0.2010
*p* value	0.2622	0.4308	0.2414	0.4164	0.4691	0.0647	0.2858	0.0874	0.3486	0.2003	0.2003	0.0844	0.3351	0.1391

In every cell, the top row indicates the Spearman's correlation coefficient (ρ), and the bottom row indicates the significance.

## Discussion

This study suggests that women who are BCS manifest cardiac autonomic modifications and HRV pattern changes compared to women with no-cancer diagnosis. BCS were characterized by a reduction of average RR interval and cardiac parasympathetic activity, a modified balance between long- and short-term HRV, lower irregularity of RR time series, and distinct fractal-like behavior. Moreover, a higher amount of visceral fat is likely manifested in BSC compared to control women. Our discussion is structured into three main findings, (1) the changes in the linear and nonlinear measures between BCS and Control women; (2) the differences in body composition in these groups, and (3) the significant correlations between HRV measures and body composition.

Concerning the linear HRV indexes, we found lower RR_ave_ values (associated with higher average heart rate) in BCS compared to controls. Generally, changes in RR intervals have been reported in BCS women even 18 years after different adjutant treatments of early breast cancer^52^. Recent results indicate that elevated heart failure risks have been observed after treatment with anthracyclines and trastuzumab treatment^53^.

Our results showed a higher cSD2/SD1 ratio in BCS women compared to Controls. Mainly, SD2/SD1 ratio offers information on the relationship between long- and short-term HRV; a higher ratio may reflect a decrease in SD1, an increase in SD2, or both. Following the interpretation of diverse authors for the SD2/SD1 ratio^55,38,54^, a higher ratio could imply a decreased vagal tone and increased sympathetic influence in BSC than Controls.

On the other hand, symbolic analysis results showed a significant decrease of the 2UV index that proves a diminished cardiac parasympathetic activity in BCS^46^. It is crucial to consider the importance of the symbolic analysis because these indexes do not suffer the heart rate bias than the linear indexes. Furthermore, a lower irregularity (indicated by SampEn) and different fractal-like behavior of the RR time series (indicated by α_2_) was found in BCS compared to Control. Lower values of SampEn have been associated with pro-inflammatory processes (e.g., experimental endotoxemia and neonatal sepsis) in preclinical and clinical studies^56,57^. Evidence indicates that fatigue is associated with a maladaptive autonomic profile characterized by lower parasympathetic activity in cancer survivors. Thus, we speculate that a diminished parasympathetic activity may be reflected as a disrupted cholinergic anti-inflammatory pathway^58^, resulting in higher inflammation in BCS. There are emerging findings on inflammatory cytokines' role in the recurrence of breast cancer^59^.

Concerning body composition, interestingly, only the nVFR was different between the BCS and cancer-free women. This result is in line with a previous study in a sample of Iranian women, indicating that most BCS are abdominally obese^6^. Another study also demonstrated a high incidence of abdominal obesity among BCS from Malaysia^60^. According to some studies, fat gain is most common for women who undergo menopause because of cancer therapy and is often accompanied by changes in body composition^61^. Relevant findings suggest that abdominal adiposity could adversely affect the sympathetic and parasympathetic function^62^. Other findings suggest that an excess of visceral fat is associated with sympathetic activation^63^.

According to the consulted literature, no studies have been conducted for evaluating the associations between central adiposity and linear and nonlinear indexes of HRV in BCS. In this study, we observed significant negative associations between visceral adiposity measured by BIA and cardiac parasympathetic function at BCS (indicated by cRMSSD, pNN50, 2UV, HFnu, cHF, and cSD1) and global heart rate variability (indicated by cSDNN). Moreover, we found significant positive associations between visceral adiposity and sympathetic function (indicated by 0V), fractal scaling properties (indicated by α_1_ and α_2_), and the balance between long- and short-term HRV (indicated by cSD2/SD1 ratio). These results are consistent with previous studies that demonstrate that central adiposity influences cardiac autonomic modulation of obese persons, increasing the risk of cardiovascular diseases^64^. Thus, we consider that a concomitant increase of visceral fat, among other factors, may contribute to cardiac autonomic modifications and changes in HRV patterns in women who are BCS.

This study found significant correlations between nVFR and HRV measures only in BCS (but not in Controls). It could be explained by the relevant role of intra-abdominal fat to influence cardiac autonomic disturbances in this specific condition, given that nVFR was the only significantly different body composition measure between the two groups. It is also essential to take into account the possibility that the ANS alterations on BCS also may be related to additional mechanisms not exclusively related to visceral fat (e.g., reduced number of control mechanisms regulating sinus node activity, loss in the exchange of information between cardiovascular subsystems, or to increased activity of one of these subsystems^65^ indicated by SampEn and α_2_).

The HRV and VFR are promising tools for evaluating the ANS and body composition in BCS, respectively. These low-cost, affordable, and non-invasive tools can be routinely monitored in BCS for a clinical assessment or control of exercise training. Exercise training has mainly shown increment muscle strength, endurance, flexibility, decreased body fat percentage, waist circumference, and visceral fat area in cancer survivors^66^. Alternative therapies such as massages^67^ and mindfulness^68^ lead to an immediate increase of HRV, reduce inflammation, and improve BCS mood with cancer-related fatigue.

### Limitations

A small sample size limits this preliminary study; our findings and interpretation should be confirmed in further clinical explorations with a larger population. Nonetheless, previous studies have confirmed that even with a small number of participants (N = 15), it is possible to detect significant differences in some indexes of HRV of BCS^27^. We recognize that Poincaré indexes and symbolic dynamics are superior that linear indexes to identify autonomic-related changes in BCS.

Additionally, the beat-to-beat change seen in the RR time series is not only under ANS influence. Humoral factors and respiratory variation may also play a role. Given the study's cross-sectional nature, it is unknown whether some observed differences had already been present before breast cancer treatment. At this stage, it is not possible to determine the causality between autonomic cardiac alterations and visceral fat. However, according to our results, we suggest that visceral fat and autonomic disturbances are highly related to BCS.

Although the decrease in parasympathetic function is mild in BCS than in Controls (2UV, p = 0.03), the BCS participants have, on average, four years after completion of chemotherapy, surgery, or medications. Thus, BCS women may be long-term repercussions in their autonomic activity, and it is likely related to a chronic inflammation process reported in BCS^69^.

In this study, we selected a simple method for correcting the HRV indexes, however, it is important to mention that some other studies have already explored new robust methods to attenuate the mean heart rate bias in linear and nonlinear HRV indexes^70,71^. We will explore these relevant methods in future work as well as studying other indexes such as fatigue, stress, or anxiety, which are shown to induce changes in HRV^72,73^ and might help explain the differences observed between the groups. Additional future work will involve applying the ECG-Derived respiration technique and the quantification of cardio-respiratory coupling in BCS data.

## Conclusions

Women who are BCS exhibited changes in HRV compared to controls; it included: (a) lower average RR intervals, (b) lower parasympathetic cardiac activity (as indicated by 2UV), a modified balance between long- and short-term HRV, and a lower irregularity of RR time series (indicated by cSD2/SD1 and SampEn, respectively) and different fractal-like behavior (indicated by α_2_). Additionally, survivors may have an increase in visceral fat compared to control women. Thus, a concomitant increase of visceral fat, among other factors, may contribute to cardiac autonomic disturbances and changes in HRV patterns in BCS. The visceral fat and HRV may be valuable biomarkers for monitoring breast cancer survivors' health and well-being.
